# MiR-146b-5p/SEMA3G regulates epithelial-mesenchymal transition in clear cell renal cell carcinoma

**DOI:** 10.1186/s13008-023-00083-w

**Published:** 2023-03-07

**Authors:** Mengxi Tang, Tao Xiong

**Affiliations:** 1 Urinary Surgery, The People’s Hospital of Rongchang District, Chongqing, 402460 China; 2 Urinary Surgery, The People’s Hospital of Rongchang District, No.3, North Square Road, Changyuan Subdistrict, Chongqing, 402460 China

**Keywords:** SEMA3G, miR-146b-5p, Metastasis, Epithelial-mesenchymal transition, Clear cell renal cell carcinoma

## Abstract

**Objective:**

The primary purpose was to unveil how the miR-146b-5p/SEMA3G axis works in clear cell renal cell carcinoma (ccRCC).

**Methods:**

ccRCC dataset was acquired from TCGA database, and target miRNA to be studied was further analyzed using survival analysis. We performed miRNA target gene prediction through the database, and those predicted miRNAs were intersected with differential mRNAs. After calculating the correlation between miRNAs and mRNAs, we completed the GSEA pathway enrichment analysis on mRNAs. MiRNA and mRNA expression was examined by qRT-PCR. Western blot was introduced to detect SEMA3G, MMP2, MMP9 expression, epithelial-mesenchymal transition (EMT) marker proteins, and Notch/TGF-β signaling pathway-related proteins. Targeted relationship between miRNA and mRNA was validated using a dual-luciferase test. Transwell assay was employed to assess cell migration and invasion. Wound healing assay was adopted for evaluation of migration ability. The effect of different treatments on cell morphology was observed by a microscope.

**Results:**

In ccRCC cells, miR-146b-5p was remarkably overexpressed, yet SEMA3G was markedly less expressed. MiR-146b-5p was capable of stimulating ccRCC cell invasion, migration and EMT, and promoting the transformation of ccRCC cell morphology to mesenchymal state. SEMA3G was targeted and inhibited via miR-146b-5p. MiR-146b-5p facilitated ccRCC cell migration, invasion, morphology transforming to mesenchymal state and EMT process by targeting SEMA3G and regulating Notch and TGF-β signaling pathways.

**Conclusion:**

MiR-146b-5p regulated Notch and TGF-β signaling pathway by suppressing SEMA3G expression, thus promoting the growth of ccRCC cells, which provides a possible target for ccRCC therapy and prognosis prediction.

**Supplementary Information:**

The online version contains supplementary material available at 10.1186/s13008-023-00083-w.

## Introduction

Clear cell renal cell carcinoma (ccRCC) is a predominant histology of RCC, occupying 75% of RCC patients [1]. Distant metastases have been observed in 25–30% of new cases with ccRCC each year [2]. Changes in the microenvironment that entails epithelial-mesenchymal transition (EMT), such as inflammation, angiogenesis, cancerous stroma, and vascular intercalation, control the progression of cancer metastasis [3]. EMT has also been linked to the pathogenesis of ccRCC [4, 5]. Therefore, studying the underlying molecular mechanisms of ccRCC metastasis and EMT is beneficial for the precise treatment of ccRCC. MicroRNAs (miRNAs) act as regulators in metastasis and EMT phenotypes of cancer cells [6, 7], so we invesgated miRNAs in this study.

As one of the subtypes of non-coding RNAs, miRNAs can specifically recognize and bind to 3ʹ-untranslated region (3ʹ-UTR) of downstream target mRNAs, facilitating mRNA degradation and impeding target gene translation [8]. MiR-146b-5p can play a pro-cancer or anti-cancer role in cancer progression. Shi et al. [9] showed that miR-146b-5p could promote colorectal cancer development through targeting TRAF6. Ren et al. [10] disclosed that miR-146b-5p is a cholangiocarcinoma inhibitor through targeting TRAF6 to repress proliferation and stimulate apoptosis of cholangiocarcinoma cells. Notably, Zhang et al. [11] proved that LINC01535/miR-146b-5p/TRIM2 axis could regulate ccRCC progression via the PI3K/Akt signaling pathway. At present, limited research has focused on molecular modulatory mechanism of miR-146b-5p in ccRCC, and the correction between miR-146b-5p and ccRCC  still has great research. Here, miR-146b-5p was taken as the target gene to study its regulatory mechanism in ccRCC development and the downstream regulatory gene SEMA3 was found.

Class 3 semaphorins (SEMA3) is a  secreted glycoproteins, approximately 100 kDa consisting of seven isoforms (SEMA3A-G), which is linked to the development of nervous system and axon guidance [12]. The role of SEMA3 in multiple effectors signaling has been found to regulate the formation of dermal lymphatic networks negatively [13]. Regarding SEMA3G, research discovered that PPAR-γ could enhance the migration of endothelial cell by promoting SEMA3G expression [14]. In addition, in a Nrp2/PlexinD1-dependent manner, SEMA3G stimulates distribution of lymphatic endothelial cells away from arteries and generates a branching network during arterial lymphatic development [15]. At present, it has been predicted that SEMA3G can be used as a prognostic indicator in ccRCC [16, 17], but there is no relevant report on the mechanism of SEMA3G in ccRCC. Our investigation found that miR-146b-5p inhibited the expression of SEMA3 expression and affected EMT in ccRCC, and it has been reported that EMT is associated with the Notch and TGF-β signaling pathways [18], so we added contents related to the Notch and TGF-β signaling in our study.

Notch receptor comprises an intracellular domain and an extracellular domain with nuclear localization motifs. Evidence suggests that Notch activity dysregulation is linked to cancer development like breast cancer [19], ovarian cancer [20], prostate cancer [21], and colorectal cancer [22]. TGF-β signaling is the most well-studied mechanism for inducing EMT, and it works via a variety of intracellular messengers. TGF-β superfamily ligands, which contain 3 TGF isoforms (TGF1, 2, and 3) and 6 BMP isoforms, generally activate signaling (BMP2 to BMP7). TGF-β signaling pathway has a connection with the development of cancers, including esophageal squamous cell carcinoma [23], liver cancer [24], prostate cancer [21], breast cancer [25], and small cell lung cancer [26]. We evaluated the impacts of miR-146b-5p and SEMA3G on the Notch and TGF-β signaling pathways in ccRCC.

This research aimed to clarify how miR-146b-5p and SEMA3G affected the modulation of Notch and TGF-β signaling, thus identifying their roles in ccRCC development. We experimentally discovered that miR-146b-5p enhanced cell growth in ccRCC cells by down-regulating SEMA3G expression and regulating the Notch and TGF-β signaling pathways.

## Materials and methods

### Bioinformatics

Expression data of ccRCC mRNAs (normal: 72, tumor: 539) and mature miRNAs (normal: 71, tumor: 545) were retrieved from TCGA database (https://portal.gdc.cancer.gov/). The R package “edgeR” (log|FC|> 1.5, FDR < 0.05) was employed to compare the expression of miRNA and mRNA in the normal and tumor groups (log|FC|> 1.5, FDR < 0.05). And we utilized R package “survival” to examine association between miR-146b-5p and the prognoses of ccRCC patients. To determine miRNA downstream regulatory target genes, we used the miRDB (http://mirdb.org/), TargetScan (http://www.targetscan.org/vert_72/), starBase (http://starbase.sysu.edu.cn/) and mirDIP (http://ophid.utoronto.ca/mirDIP/index.jsp#r) databases. Differential mRNAs with targeted binding sites to target miRNAs were screened, which were intersected with differentially down-regulated mRNAs. Then target genes were finally determined using correlation analysis. GSEA software was applied to perform a KEGG analysis.

### Cell culture

BeNa Culture Collection (BNCC, China) provided human normal renal cell lines HK-2 (BNCC339833), ccRCC cell lines 786-O (BNCC338472), 769-P (BNCC341606), Caki-2 (BNCC340136) and renal carcinoma cell line A498 (BNCC338630). Cells were cultivated in a 5% CO_2_ incubator at 37 ℃. The mediums used for each cell line were as follows:

HK-2, A498, 769-P cell lines: RPMI-1640 medium (BNCC341471, BNCC, China) containing 10% fetal bovine serum (FBS);

786-O cell line: DMEM medium (BNCC351841, BNCC, China) containing 10% FBS;

Caki-2 cell line: McCoy’s 5a culture medium (BNCC341856, BNCC, China) containing 10% FBS.

### Cell transfection

MiR-146b-5p mimic (miR-mimic) and mimic NC (miR-NC), miR-146b-5p inhibitor (miR-inhibitor) and inhibitor NC (inhibitor-NC), pcDNA3.1-SEMA3G plasmid encoding SEMA3G (oe-SEMA3G), and blank pcDNA3.1 plasmid (oe-NC) vectors were all acquired from RiboBio (China). 786-O cell line was transfected with miR-inhibitor/inhibitor-NC and miR-mimic/miR-NC or pcDNA3.1-SEMA3G/blank pcDNA3.1 plasmid was transfected into Caki-2 cell line by Lipofectamine 2000 kit (Invitrogen, USA). Transfection efficiency was detected after 24 h of transfection. Transfection sequences are shown in Table 1. Additionally, to evaluate whether the transfection efficiency changed with transfection time, we further explored the transfection efficiency after 48 h and 72 h.

**Table 1:** Transfection sequences of genes

Gene	Sequence
miRNA-146b-5p mimic	5ʹ-UGAGAACUGAAUUCCAUAGGCU-3ʹ
mimic NC	5ʹ-UUCUCCGAACGUGUCACGUTT-3ʹ
miRNA-146b-5p inhibitor	5ʹ-AGCCUAUGGAAUUCAGUUCUCA-3ʹ
inhibitor NC	5ʹ-CAGUACUUUUGUGUAGUACAA-3ʹ
oe-SEMA3G	5ʹ-ATCTGTCTCCATGCTTTGGAAT-3ʹ
oe-NC	5ʹ-ATCTGTCTCCATGCTTTGGAAT-3ʹ

### Real-time PCR detection

TRIzol reagent (Life Technologies, USA) was the tool for extracting total RNA from cells, and RNA concentration was assayed on NanoDrop 2000 system (Thermo Fisher Scientific, USA). Hairpin-it miRNAs qRT-PCR kit (GenePharm, China) was used to reverse-transcribe miRNAs into cDNA, and PrimeScript RT Master Mix (Takara, Japan) was applied to reverse-transcribe mRNA into cDNA. MiRNA and mRNA expression were evaluated using miScript SYBR Green PCR Kit (Qiagen, Germany) and SYBR^®^ Premix Ex Taq TM II (Takara, Japan). The endogenous control for miR-146b-5p was U6, and that for SEMA3G and other mRNAs was GAPDH. Relative expression of miR-146b-5p and mRNAs was compared using the 2^−ΔΔCt^ value. Table 2 lists the primer sequences.

**Table 2:** Primer sequences used for qRT-PCR

Gene	Sequence	
miRNA-146b-5p	Forward primer	5ʹ-GGGCGGTGAGAACTGAATT-3ʹ
	Reverse primer	5ʹ-CAGTGCGTGTCGTGGAGT-3ʹ
U6	Forward primer	5ʹ-CTCGCTTCGGCAGCACA-3ʹ
	Reverse primer	5ʹ-AACGCTTCACGAATTTGCGT-3ʹ
SEMA3G	Forward primer	5ʹ-GGGTCTGTGCTCAAAGTCATCG-3ʹ
	Reverse primer	5ʹ-AAGTCCCACTGCCTCTTCTTCC-3ʹ
GAPDH	Forward primer	5ʹ-AGAAGGCTGGGGCTCATTTG-3ʹ
	Reverse primer	5ʹ-AGGGGCCATCCACAGTCTTC-3ʹ
NOTCH1	Forward primer	5ʹ-GCAGAGGCGTGGCAGACTAT-3ʹ
	Reverse primer	5ʹ-CAGTAGAAGGAGGCCACACG-3ʹ
Snail1	Forward primer	5ʹ-CTCGGACCTTCTCCCGAATG-3ʹ
	Reverse primer	5ʹ-AAAGTCCTGTGGGGCTGATG-3ʹ
E-Cadherin	Forward primer	5ʹ-AAGTTGAGCCCCAAGGTGAT-3ʹ
	Reverse primer	5ʹ-CTGGAAGGAGCGGTTCTTTTT-3ʹ
N-cadherin	Forward primer	5ʹ-CACCGACGTAGACAGGATCG-3ʹ
	Reverse primer	5ʹ-CGTCTAGCCGTCTGATTCCC-3ʹ
Vimentin	Forward primer	5ʹ-TCCTGCCAATGTTGCTCACT-3ʹ
	Reverse primer	5ʹ-CCAGCCACTGTTGTCAGAGT-3ʹ
Slug	Forward primer	5ʹ-CTCCTCATCTTTGGGGCGAG-3ʹ
	Reverse primer	5ʹ-TCCTTGAAGCAACCAGGGTC-3ʹ

### Western blot

RIPA lysis buffer (Thermo Fisher Scientific, USA) was employed to extract proteins, and protein quantification was determined by the BCA Kit (Beyotime, China). After proteins (30 µg per lane) were separated on 12% SDS-PAGE gels, they were transferred onto PVDF (Millipore, USA) membranes. Membranes were blocked for 2 h with 5% nonfat dried milk and then maintained with primary antibodies at 4 ℃ overnight. Membranes were then rinsed three times in PBST buffer for 30 min and probed with secondary antibody at room temperature for 2 h. Protein signals were detected with an enhanced chemiluminescence kit (Thermo Fisher Scientific, USA). Primary antibodies employed in this research were: rabbit anti-SEMA3G (ab197108, 1:500), rabbit anti-MMP2 (ab92536, 1:2000), rabbit anti-MMP9 (ab76003, 1:2000), rabbit anti-Snail1 (ab216347, 1:1000), rabbit anti-Snail2 (Slug, ab63568, 1:500), rabbit anti-E-cadherin (ab40772, 1:25,000), rabbit anti-N-cadherin (ab18203, 1:10,000), rabbit anti-Vimentin (ab92547, 1:3000), rabbit anti-Notch1 (ab52627, 1:1500), rabbit anti-Hes1 (ab108937, 1:1000), rabbit anti-Hes5 (ab194111, 1:1000), rabbit anti-TGFβR1 (ab235178, 1:1000), rabbit anti-TGFβR2 (ab186838, 1:1000), rabbit anti-Smad3 (ab40854, 1:5000), rabbit anti-p-Smad3 (ab52903, 1:2000) and rabbit anti-GAPDH (ab181602, 1:10,000). Secondary antibody was goat-anti-rabbit IgG (ab205718, 1:10,000). All of them were procured from Abcam (UK).

### Transwell assay

ccRCC cells (1 × 10^5^ cells) were plated into the upper chamber of the device coated with Matrigel (BD Biosciences, USA). Next, 700 μL of DMEM containing 20% FBS was filled into the lower chamber. The cells were kept routine conditions for 24 h. The non-invaded cells were removed from the filter membrane, and invaded ones were immobilized with 4% paraformaldehyde for 20 min and dyed with 0.1% crystal violet for 30 min. Photographs were taken under a microscope and cells passing through the membrane were counted. Matrigel was not applied to the upper chamber in the migration assay, and other steps were the same with the invasion assay.

### Wound healing experiment

Cells at the logarithmic growth phase (2 × 10^5^ cells/well) were plated with 6-well plates, with a volume of 2 mL per well. When the cells filled the wells, the cell layer in each well was marked with a “ + ” with a sterilized 200 μL pipette tip. When scratching, pipette tip was kept perpendicular to the bottom of the plate to ensure the scratch straight and uniform. Then cells were rinsed 4 times with PBS to wash off scratched cells. The remained cells were kept in serum-free DMEM. The wound healing condition was observed and photoed at 0 h and 24 h of incubation.

### Dual-luciferase assay

Binding of miR-146b-5p to the 3ʹ-UTR of SEMA3G was identified via dual-luciferase assay. Firstly, pmirGLO-SEMA3G-3ʹ-UTR-WT and pmirGLO-SEMA3G-3ʹ-UTR-MUT luciferase reporter vectors (Promega, USA) were established. The ccRCC cell line Caki-2 was plated in 96-well plates (2 × 10^5^ cells/well) and co-transfected with 3 µg of miR-mimic/miR NC and SEMA3G-WT/SEMA3G-MUT plasmids. Luciferase activity was measured after 48 h of culture on Dual-Luciferase Reporter System (Promega, USA).

### Analysis of statistics

All data were processed using GraphPad Prism 6.0 (La Jolla, CA) and presented as mean ± standard deviation. Two-group comparing was done using t-test. One-way ANOVA analysis of variance was used for comparison of more than three groups. All experiments were performed in 3 biological replicates. *P < 0.05 indicates statistical differencee, **P<0.01 indicates significant difference, ***P<0.001 indicates extremely significant difference.

## Result

### MiR-146b-5p is significantly highly expressed in ccRCC cells, and correlates with poor prognosis

**Fig. 1:**
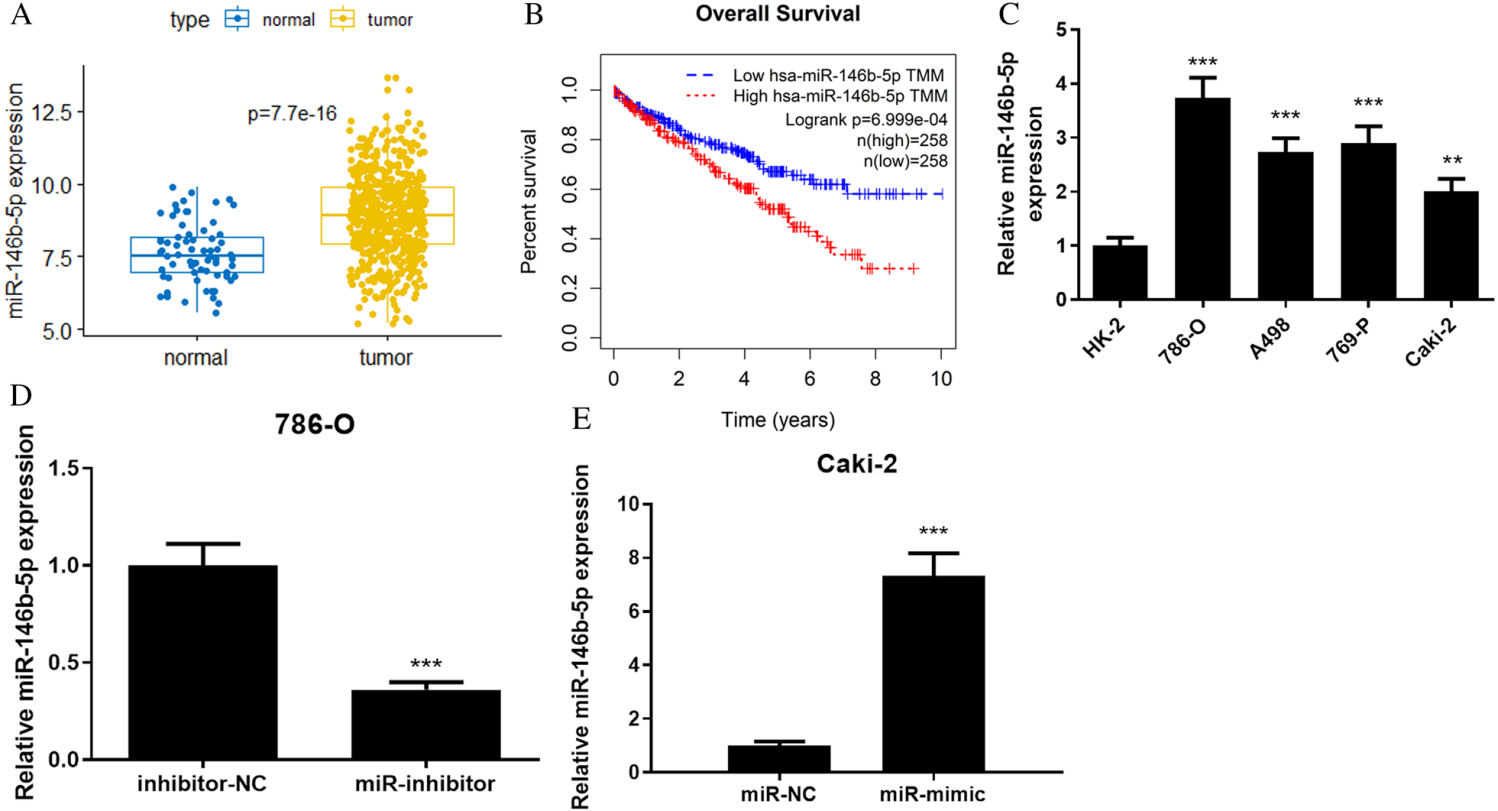
MiR-146b-5p is highly expressed in ccRCC cells. **A**: Expression of miR-146b-5p, blue box plot indicates normal samples, yellow box plot indicates tumor samples; **B**: Overall survival curve of miR-146b-5p expression level on patients, red represents high miR-146b-5p expression, blue represents low miR-146b-5p expression; **C**: Expression of miR-146b-5p in normal cell line HK-2 and ccRCC cell lines 786-O, A498, 769-P and Caki-2; **D**: Transfection efficiency of knockdown miR-146b-5p in 786-O cells; **E**: Transfection efficiency of overexpression miR-146b-5p in Caki-2 cells; All the above experiments were performed with 3 biological replicates; ** *P*  <0.01 indicates siginficant difference, ***P<0.001 indicates extremely significant difference.

Firstly, TCGA database (https://portal.gdc.cancer.gov/) was utilized to predict miR-146b-5p expression. Compared to normal renal tissues, miR-146b-5p was strikingly and strongly expressed in ccRCC tissues (Fig. 1A). Next, we used the R package “survival” to measure correlation between miR-146b-5p expression and data from 10-year follow-up of the ccRCC patients. We further performed Kaplan–Meier (K–M) survival curves to analyze the results, which showed that patients with higher expression of miR-146b-5p presented prominently shorter overall survival than those with lower miR-146b-5p expression (Fig. 1B). Based on the results of the above bioinformatics analyses, we further analyzed miR-146b-5p expression at cellular level. The results of qRT-PCR revealed that miR-146b-5p expression in ccRCC cells was much higher than in normal human renal cells. (Fig. 1C). Then, miR-146b-5p mimic or miR-146b-5p inhibitor were transfected into ccRCC cells to reveal the potential role of miR-146b-5p in ccRCC. Since miR-146b-5p expression was highest in 786-O cell line and lowest in Caki-2 cell line, the 786-O was used for knockdown treatment, and the Caki-2 was used for overexpression treatment, and transfection efficiency was examined by qRT-PCR. We evaluated transfection efficiency of miR-146b-5p mimic in Caki-2 cells or of miR-146b-5p inhibitor in 786-O cells at 24, 48, and 72 h, and the results demonstrated that the transfection efficiency at 24 h reached a good level. Therefore, the subsequent transfection time was set as 24 h (Additional file 1: Fig. S1). MiR-146b-5p expression was reduced in 786-O cells with miR-146b-5p inhibitor (Fig. 1D), which was increased in Caki-2 cells with miR-146b-5p mimic (Fig. 1E). These findings indicated that miR-146b-5p was significant high-expressed in ccRCC cells and correlated with poor prognosis.

### MiR-146b-5p enhances migration, invasion and EMT of ccRCC cells

**Fig. 2:**
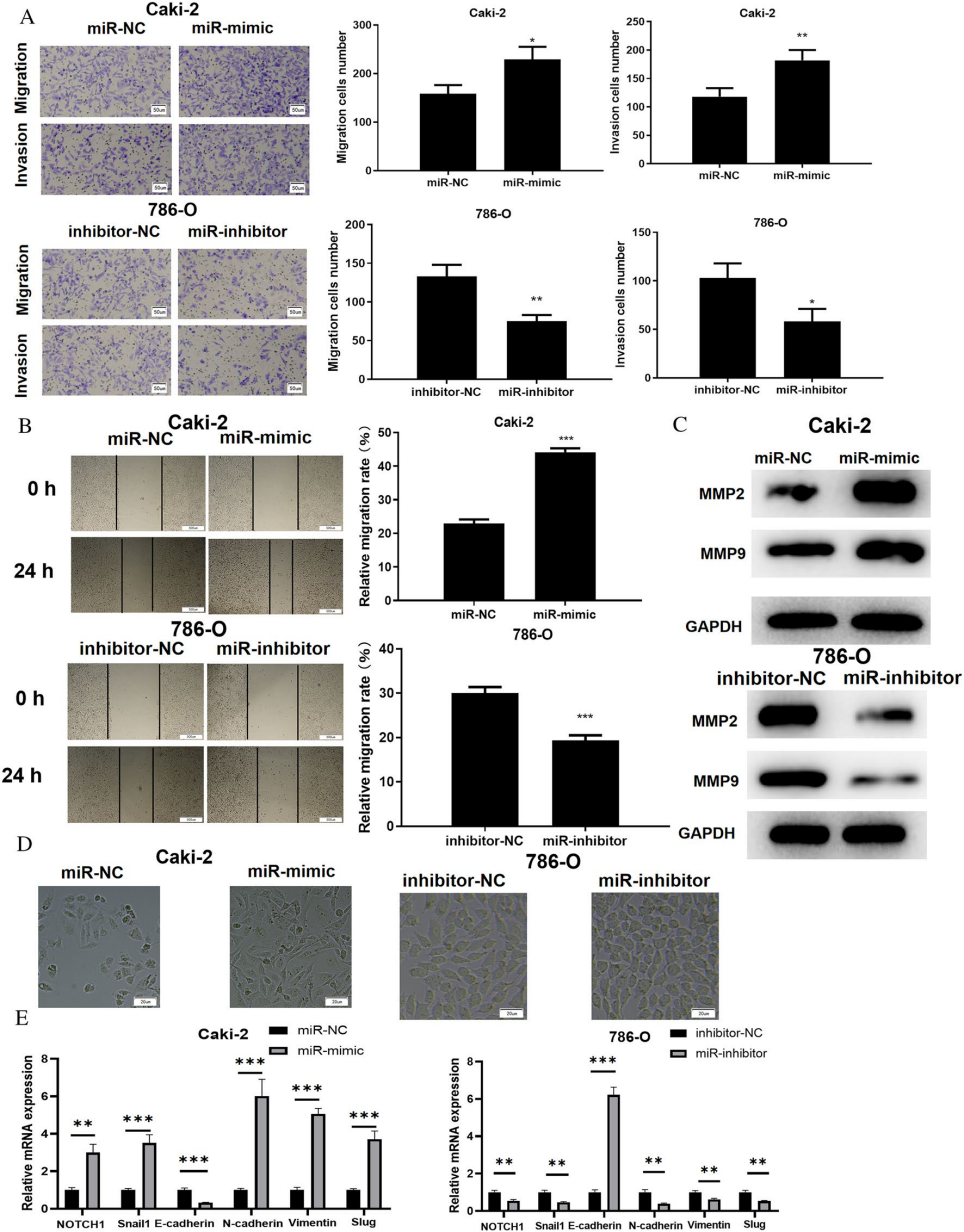

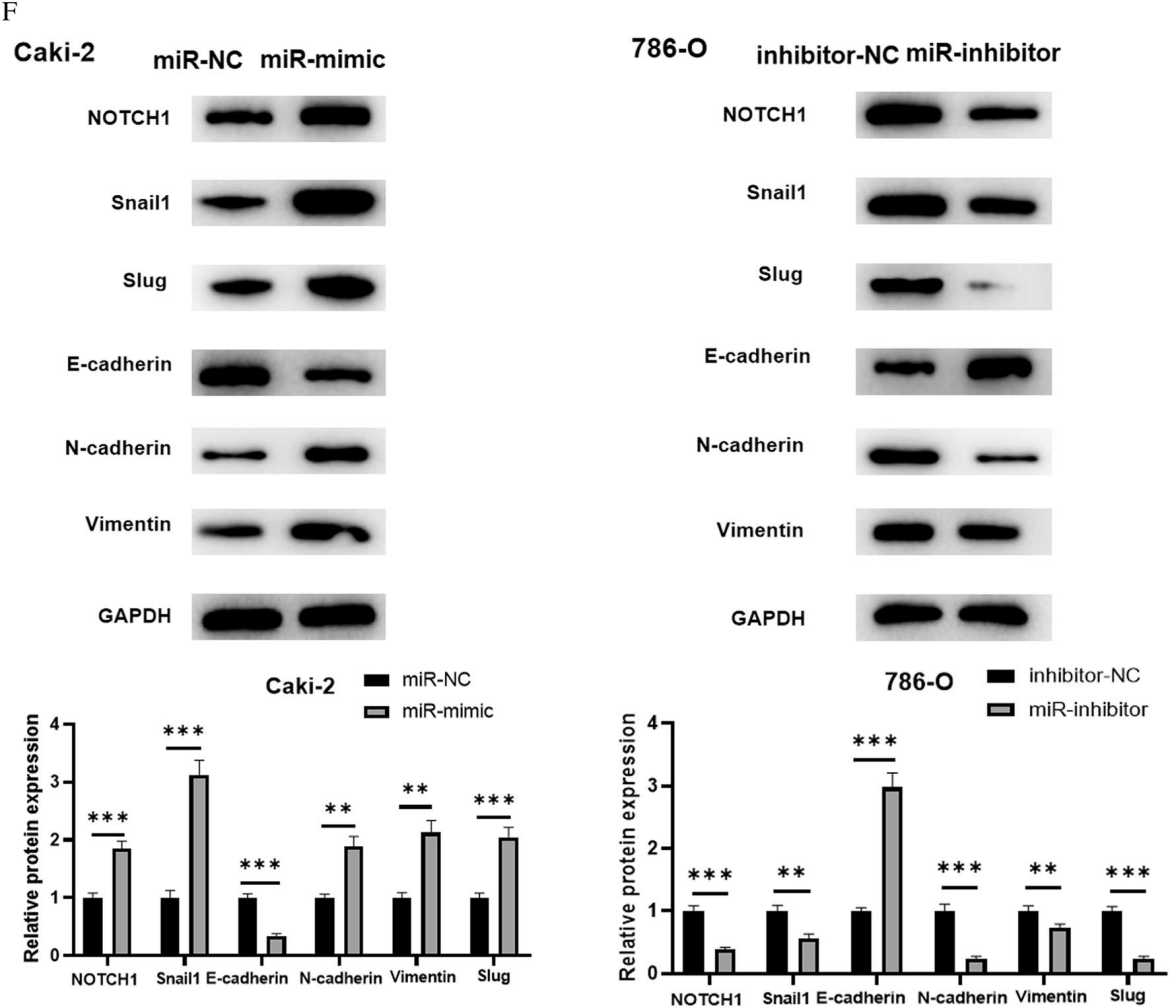
MiR-146b-5p promotes migration, invasion and EMT of ccRCC cells. **A**, **B**: Transwell assay and wound healing experiment were employed to determine the migratory and invasive abilities of ccRCC cells 786–O and Caki-2 in different treatment groups (100 ×); **C**: Western blot assay was employed to measure the expression levels of MMP2 and MMP9 in 786–O and Caki-2cells in different treatment groups; **D**: The morphology of 786–O and Caki-2 cells in each transfection group was observed under microscope; **E**: qRT-PCR evaluated the mRNA levels of Notch1, Snail1, Snail2, E-cadherin, N-cadherin and Vimentin in 786–O and Caki-2 cells; **F**: The protein expression of Notch1, E-cadherin, N-cadherin, Vimentin, Snail1, Snail2, and GAPDH in ccRCC cells 786–O and Caki-2 in different treatment groups; All the above experiments were performed with 3 biological replicates; * *P* < 0.05 indicates statistical difference, **P<0.01 indicates significant difference, ***P<0.001 indicates extremely siginficant difference.

It has been validated that abnormal expression of miR-146b-5p is implicated in progression of malignancies [27–29]. Next, we explored the modulatory role of miR-146b-5p in migration, invasion and EMT of ccRCC cells. We used transwell test and wound healing experiment to assess cell migration and invasion. The findings illustrated that knocking down miR-146b-5p markedly decreased ccRCC cell migration and invasion, whereas overexpressing miR-146b-5p boosted ccRCC cell migration and invasion considerably (Fig. 2A, B). MMP2 and MMP9 are strongly expressed in varying malignant tumor tissues and are strongly associated with tumor cell invasion and metastasis [30]. Therefore, we analyzed the expression of MMP2 and MMP9 in ccRCC cells via western blot assay, which showed that miR-146b-5p knockdown evidently suppressed expression of MMP2 and MMP9 in ccRCC cells. However, overexpression of miR-146b-5p evidently enhanced the levels of MMP2 and MMP9 (Fig. 2C), which confirmed the experimental results in Fig. 2A, B. Many mechanistic investigations have discovered that elevated miR-146b-5p expression can trigger EMT and play a tumor-promoting or tumor-suppressing role [31, 32]. Therefore, we looked in whether miR-146b-5p could influence EMT progression of ccRCC cells. Firstly, we analyzed the cell morphology of ccRCC cells under the microscope, which presented that miR-146b-5p mimic treatment enhanced Caki-2 cell adhesion and polarity, reduced pseudopodia formation. While the changes after miR-146b-5p inhibitor treatment in 786-O cells were opposite (Fig. 2D). In addition, Snail1 and Snail2 are important transcription factors in EMT and are closely related to Notch signaling pathway [33–35]. Therefore, when detecting mRNA and protein expression levels of EMT markers using qRT-PCR and western blot, the expression levels of Snail1, Snail2 (Slug) and Notch1 were also detected. qRT-PCR and western blot unraveled that knocking down miR-146b-5p elevated the expression of EMT marker protein E-cadherin but reduced the expression of Notch1, Snail1, Snail2, N-cadherin and Vimentin in ccRCC cells, suggesting that EMT process of ccRCC cells was hindered. Increased miR-146b-5p had the opposite results, indicating that overexpressed miR-146b-5p promoted EMT process of ccRCC cells (Fig. 2E, F). Taken together, the above results suggested that miR-146b-5p could facilitate migration, invasion, and EMT of ccRCC cells.

### MiR-146b-5p targetes and down regulates SEMA3G expression

**Fig. 3:**
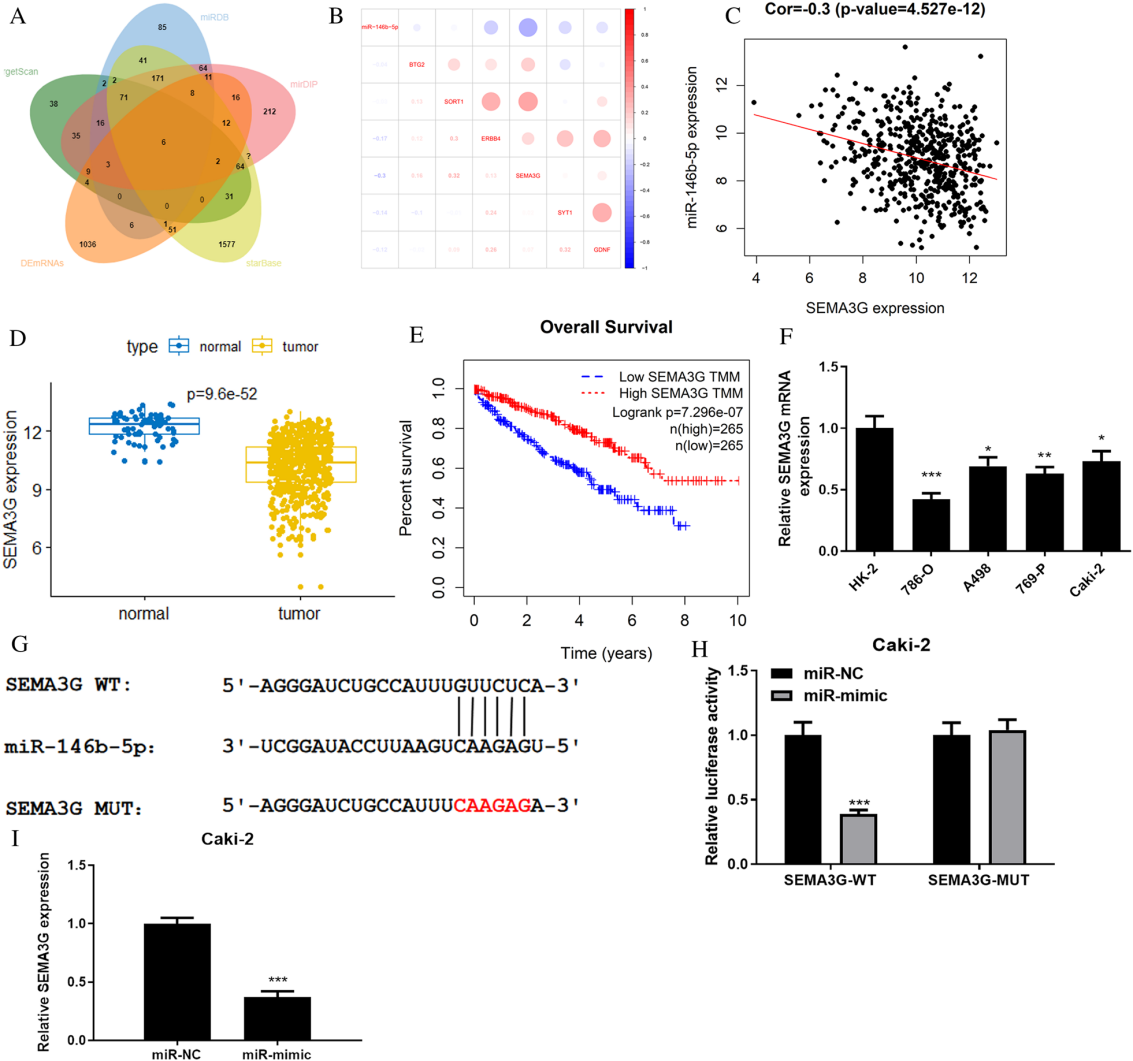
MiR-146b-5p targets the expression of SEMA3G **A**: Venn diagram of miR-146b-5p predicted target mRNA and down-regulated differential mRNAs. Green indicates TargetScan database, orange indicates down-regulated differential mRNAs in TCGA-KIRC dataset, yellow indicates starBase database, pink indicates mirDIP database, blue indicates miRDB database; **B**: Pearson correlation analysis plot of miR-146b-5p and candidate genes; **C**: Pearson correlation analysis plot of miR-146b-5p and SEMA3G; **D**: Expression of SEMA3G in TCGA-KIRC database, blue box plot indicates normal samples, yellow box plot indicates tumor samples; **E**: Expression level of SEMA3G on overall survival curve of patients. Red indicates high expression of SEMA3G. Blue represents low SEMA3G expression; **F**: SEMA3G mRNA expression level in normal cell line HK-2 and ccRCC cell lines 786–O, A498, 769-P and Caki-2; **G**: Schematic diagram of SEMA3G-WT and SEMA3G-MUT binding to miR-146b-5p sequence predicted by starBase; **H**: Dual-luciferase assay was used to detect luciferase activity in different treatment groups in ccRCC cell line Caki-2; **I**: qRT-PCR was performed to detect the regulatory relationship between miR-146b-5p and SEMA3G in Caki-2 cells; All the above experiments were performed with 3 biological replicates; *P < 0.05 indicates statistical difference, **P<0.01 indicates significant difference, ***P<0.001 indicates extremely significant difference.

To unravel the modulatory mechanism of miR-146b-5p downstream in ccRCC cells, we performed the differential analysis of mRNAs in TCGA-KIRC database. We intersected differentially down-regulated mRNAs with miR-146b-5p target genes predicted through TargetScan, miRDB, mirDIP, and starBase databases, and obtained 6 differential mRNAs which had targeted binding sites with miR-146b-5p (Fig. 3A). Next, we performed Pearson correlation analysis on the 6 predicted differential mRNAs and miR-146b-5p. Pearson correlation analysis revealed that miR-146b-5p had the strongest negative connection with SEMA3G (Fig. 3B, C), and the role of SEMA3G in the malignant progression of ccRCC had not been reported yet. Therefore, SEMA3G was chosen as the research subject. Next, we conducted bioinformatics analysis on SEMA3G, and data from TCGA-KIRC database (normal: 72, tumor: 539) showed that the expression level of SEMA3G in ccRCC tissues was remarkably lowerthan that  in normal tissues (Fig. 3D). Subsequently, bioinformatics R package “survival” was applied to analyze correlation between SEMA3G expression and the prognosis of ccRCC patients. K-M analysis demonstrated that patients with low SEMA3G level had a much shorter survival than those with high level (Fig. 3E). Next, the above bioinformatics analysis results were verified by cell functional experiments. qRT-PCR exhibited that the mRNA level of SEMA3G was substantially reduced in ccRCC cells (Fig. 3F), in line with the above bioinformatics analysis results. Whereafter, we predicted a binding site for miR-146b-5p with SEMA3G through bioinformatics analysis (Fig. 3G). Dual-luciferase assay further confirmed the targeted relationship between miR-146b-5p and SEMA3G. Overexpression of miR-146b-5p inhibited wild-type SEMA3G 3’-UTR luciferase activity, but did not affect mutant SEMA3G 3’-UTR luciferase activity, which indicated that miR-146b-5p could target and bind to SEMA3G 3’-UTR (Fig. 3H). Finally, qRT-PCR analyzed the regulatory relationship between miR-146b-5p and SEMA3G. Experimental findings illustrated that overexpression of miR-146b-5p could repress SEMA3 expression (Fig. 3I). Accordingly, we concluded that SEMA3G was a target of miR-146b-5p and downregulated by miR-146b-5p.

### MiR-146b-5p targets SEMA3G to modulate Notch and TGF-β signaling, thereby affecting migration, invasion and EMT of ccRCC cells

**Fig. 4:**
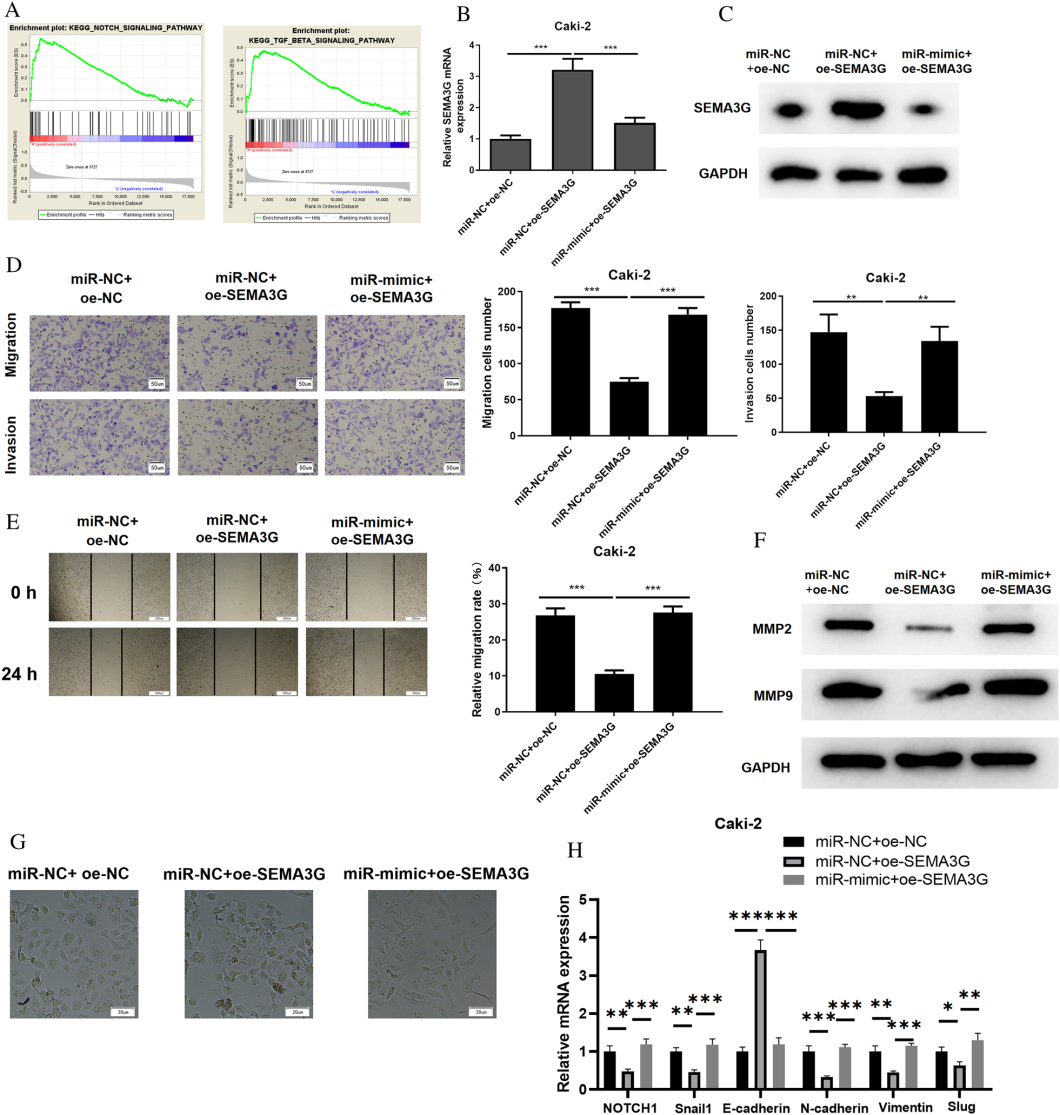

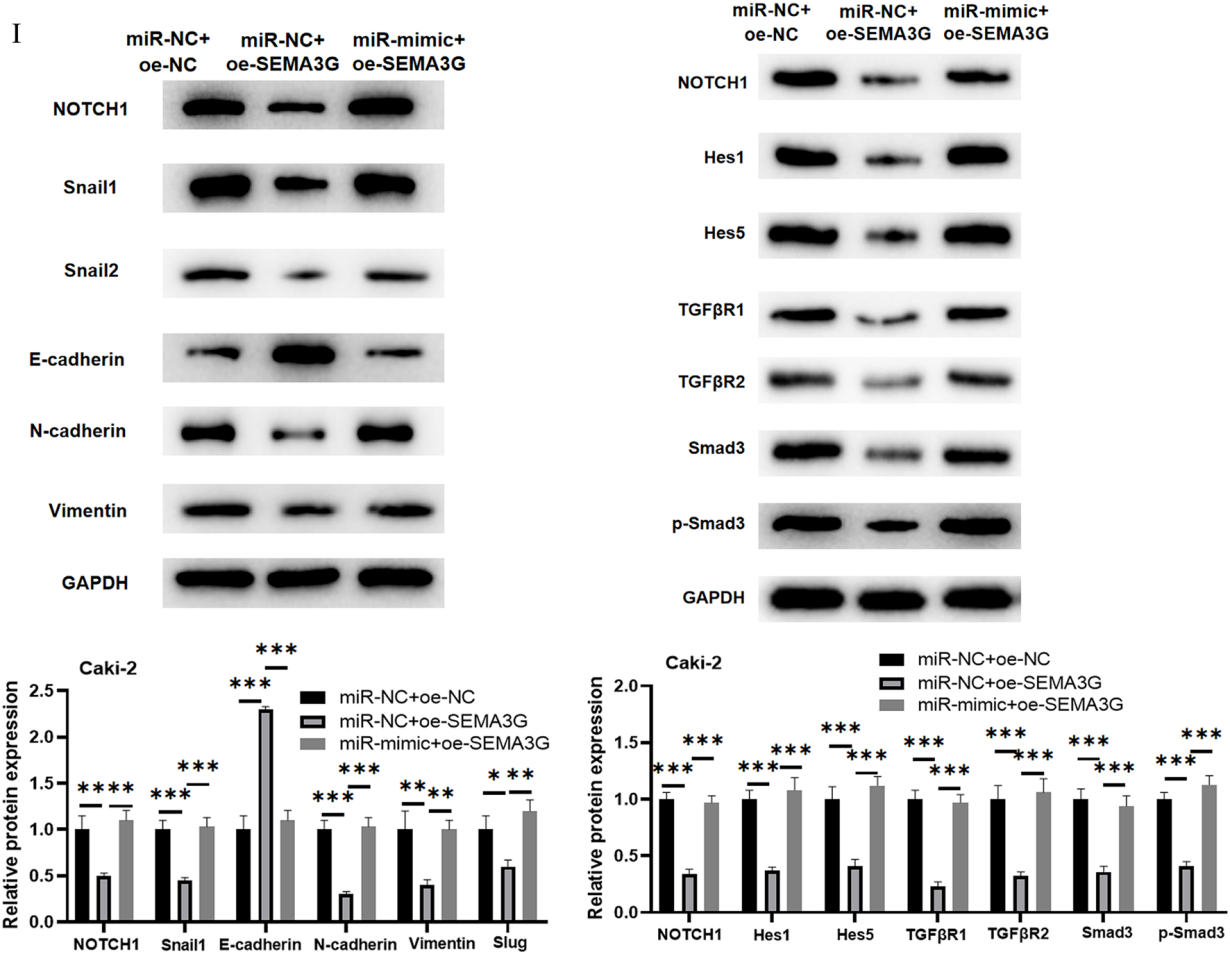
MiR-146b-5p targets SEMA3G to regulate Notch and TGF-β signaling pathways to affect the migration, invasion and EMT of ccRCC cells. **A**: GSEA pathway enrichment analysis results of SEMA3G; **B**, **C**: The mRNA and protein expression of SEMA3G in different treatment groups of ccRCC cells Caki-2; **D**, **E**: Transwell assays and wound healing experiment were performed to inspect the migratory and invasive abilities of ccRCC cells Caki-2 in different treatment groups (100 ×); **F**: Western blot was performed to inspect the expression levels of MMP2 and MMP9 in Caki-2 cells; **G**: The morphology of Caki-2 cells in each transfection group was observed under microscope; **H**: qRT-PCR evaluated the mRNA levels of Notch1, Snail1, Snail2, E-cadherin, N-cadherin and Vimentin in Caki-2 cells; **I**: The expression of EMT marker proteins, Notch, and TGF-β signaling pathway marker proteins in different treatment groups of ccRCC cells Caki-2; All the above experiments were performed with 3 biological replicates; *P < 0.05 indicates statistical difference, **P<0.01 indicates significant difference, ***P<0.001 indicates extremely significant difference. One-way ANOVA analysis of variance was used for comparison of three groups.

In the above studies, we have proved stimulative impacts of miR-146b-5p on ccRCC cell migration and invasion. Here, we studied effects of miR-146b-5p/SEMA3G on functions of ccRCC cells. Firstly, we conducted GSEA for SEMA3G and found that SEMA3G was mainly concentrated in the Notch and TGF-β signaling (Fig. 4A). To investigate whether miR-146b-5p/SEMA3G modulates ccRCC cell phenotypes through the Notch and TGF-β signaling, we constructed a SEMA3G overexpression cell line (miR-NC + oe-SEMA3G) and a simultaneous overexpression cell line of miR-146b-5p and SEMA3G (miR-mimic + oe-SEMA3G) using Caki-2 cell line. As evaluated by qRT-PCR and western blot, mRNA and protein expression levels of SEMA3G were elevated in miR-NC + oe-SEMA3G group. Compared to miR-NC + oe-SEMA3G group, SEMA3G mRNA and protein levels were repressed in cells after overexpressing both miR-146b-5p and SEMA3G. (Fig. 4B, C). MiR-146b-5p attenuated SEMA3G expression, and that these cells could be utilized for subsequent assays. Afterward, we performed transwell assays and wound healing experiment, and discovered that cancer cell migration and invasion were significantly reduced following SEMA3G overexpression. However, when miR-146b-5p and SEMA3G were overexpressed at the same time, migratory and invasive abilities of cancer cells were considerably increased in contrast to those with SEMA3G overexpression alone (Fig. 4D, E). Western blot measured the expression of MMP2 and MMP9. According to the results, the expression levels of MMP2 and MMP9 were significantly decreased in miR-NC + oe-SEMA3 group compared to miR-NC + oe-NC group, while those in miR-mimic + oe-SEMA3G group returned to the levels in miR-NC + oe-NC group (Fig. 4F). As for the results of cell morphology analysis, in comparison with miR-NC + oe-NC group, miR-NC + oe-SEMA3 group revealed loss of intercellular adhesion, loss of cell polarity and increased pseudopodia formation, while the cell morphology of miR-mimic + oe-SEMA3G group was similar to that of miR-NC + oe-NC group (Fig. 4G). mRNA expression levels of Notch1, Snail1, Snail2 and EMT markers E-cadherin, N-cadherin and Vimentin were evaluated by qRT-PCR (Fig. 4H) . Notch and TGF-β signaling pathway-related proteins Notch 1, Hes1, Hes5, TGFβR1, TGFβR2, Smad3, and p-Smad3 in cells of different treatment groups were then determined using western blot (Fig. 4 I). EMT process and the activities of Notch and TGF-β signaling pathways were inhibited in ccRCC cells during SEMA3G overexpression, but after simultaneous increasing miR-146b-5p and SEMA3G, EMT process and activities of the Notch and TGFβ signaling pathways in ccRCC cells were notably increased compared with those when SEMA3G was overexpressed alone. All in all, the results above demonstrated that miR-146b-5p facilitated migration, invasion, and EMT of tumor cells by down-regulating SEMA3G via the Notch and TGF-β signaling pathways.

## Discussion

MiRNAs are novel and prospective biomarkers for diagnosing and treating human malignancies. MiRNAs’ role and regulation mechanism in human cancers has been a research hotspot in recent years. MiR-146b-5p dysregulation is unveiled in many cancers. For example, miR-146b-5p controls colorectal cancer cell proliferation, invasion, and metabolism targeting PDHB [29]. Through targeting ZNRF3, miR-146b-5p facilitates thyroid cancer metastasis and triggers EMT [32]. The results of bioinformatics analysis in the current study showed that miR-146b-5p expression was considerably up-regulated in ccRCC cells, which was confirmed by cell biological assays and consistent with the study of Zhang et al [11]. Herein, cell functional experiments unraveled that miR-146b-5p enhanced ccRCC cell migration, invasion, and EMT, which also proved correlation between miR-146b-5p and EMT and agreed with previous study [32].

In this study, a bioinformatics investigation revealed SEMA3G as a new putative target gene downstream of miR-146b-5p. Dual luciferase assay was adopted to validate binding relationship between miR-146b-5p and SEMA3G. There are few reports on the role of SEMA3G as an oncogene, and SEMA3G prevents tumor cell migration and invasion in gliomas [36]. Li et al. [37] disclosed that low level of SEMA3G is a protective factor in ccRCC. According to the results of bioinformatics analysis as well as the cell experiments in the current study, SEMA3G was downregulated in ccRCC cells, which agreed with Li et al. Rescue assay has illustrated that miR-146b-5p accelerated ccRCC cell migration, invasion and EMT by inhibiting SEMA3G expression.

In addition, this study also discovered that SEMA3G was enriched in Notch and TGF-β signaling pathways, thereby regulating cellular EMT progression. Some studies have confirmed the close association of these two signaling pathways with EMT. Previous studies have found that Notch signaling can regulate SNAI1 expression directly [38, 39] or indirectly through inducing hypoxia-inducible factor 1α (HIF-1α). Interaction of Snail2 with Notch is crucial for Notch-mediated E-cadherin inhibition and β-catenin activation in mouse mammary epithelial to mesenchymal (NMuMG) cells [40, 41]. Notch overexpression in endothelial cells gives rise to the loss of vascular endothelial-cadherin, leading to endothelial transition (EndMT) [38]. Inhibited Notch1 decreased cell invasiveness and partially reversed EMT in lung adenocarcinoma cells [42]. We measured the expression of Notch1 and Snail1 in ccRCC cells and found that miR-146b-5p/SEMA3G axis could facilitate the expression of Notch1 and Snail1. Moreover, detection of EMT-related markers confirmed that miR-146b-5p/SEMA3G axis fostered EMT, which confirmed the correlation between Notch1, Snail1 and EMT, echoing previous studies. TGF-β signaling is of essence for maintaining immune responses and tissue homeostasis [43, 44]. It plays a role in multiple cellular functions associated with the environment, such as differentiation, growth arrest, proliferation, EMT and apoptosis [45–47]. Currently, most cancer and fibrotic EMT are regulated by TGFβ1 [48], while TGFβ2 primarily controls EndMT in the heart development [49], and TGFβ3 mediates EMT in development [50]. Many studies has reported the involvement of signaling pathways such as Notch [51, 52] and TGF-β [53, 54] in the biological and pathological processes of ccRCC, but there are few reports on the involvement of signaling pathways such as Notch and TGF-β regulated by miRNAs in ccRCC development. Zhang et al. [55] found that miR-154 can regulate Wnt/β-catenin and Notch activities in RCC. Aberrant activation of Notch signaling pathway is closely implicated in cell proliferation, metastasis and EMT [56]. Jingushi et al. [57] disclosed that miR-629 can promote the TGF-β/Smad signaling and accelerate ccRCC migration and invasion via TRIM33. Based on these, we looked deeply into modulatory role of miRNA-146b-5p on SEMA3G and even signaling pathways like Notch and TGF-β in ccRCC. As our study revealed, overexpression of SEMA3G reduced the activity of Notch/TGF-β signaling pathway. And it decreased migration, invasion, and EMT of ccRCC cells compared to controls, but overexpressed of miR-146b-5p reverted these effects. Therefore, we suggested that miR-146b-5p fostered migration, invasion, and EMT of ccRCC cells by inhibiting SEMA3G expression from inactivating signaling pathways such as Notch/TGF-β. These findings provided a molecular mechanism of SEMA3G on ccRCC metastasis and EMT, which supported theoretical establishment of SEMA3G as a modulator and facilitates precision treatment of ccRCC.

In conclusion, miR-146b-5p facilitated migration, invasion and EMT of ccRCC cells by downregulating SEMA3G expression and activating signaling pathways such as Notch/TGF-β. For the first time, functions of miR-146b-5p/SEMA3G axis in ccRCC migration, invasion, and EMT are shown in this work. These findings are conducive to the completion of modulatory network downstream of miR-146b-5p in ccRCC progression. We provided a new entry point to find new targeted therapeutic pathways and therapeutic ideas for ccRCC, which increased our knowledge of SEMA3G regulatory involvement in signaling networks, as well as its role in cancer development. As a matter of course, this study has some potential limitations, mainly lies in the lack of clinical examination of miR-146b-5p/SEMA3G expression, and lack of relevant functional verification from the perspective of in vivo experiments. It is believed that more progress will be made in the treatment of ccRCC with further research.

## Supplementary Information

**Additional file 1. Figure S1** Correlation between transfection efficiency and transfection time. A-B: Transfection efficiency of miR-146b-5p mimic in Caki-2 cells or of miR-146b-5p inhibitor in 786-O cells at 24 h, 48 h and 72 h; All the above experiments were performed with 3 biological replicates; ** *P*<0.01 indicates significant difference, ***P<0.001 indicates extremely significant difference.

## Data Availability

The data and materials in the current study are available from the corresponding author on reasonable request.
