# Supplementary figures and images for: MiR-146b-5p/SEMA3G regulates epithelial-mesenchymal transition in clear cell renal cell carcinoma
Source: Cell Div. 2023 Mar 7;18:4. doi: 10.1186/s13008-023-00083-w (PMC9993666; doi:10.1186/s13008-023-00083-w)

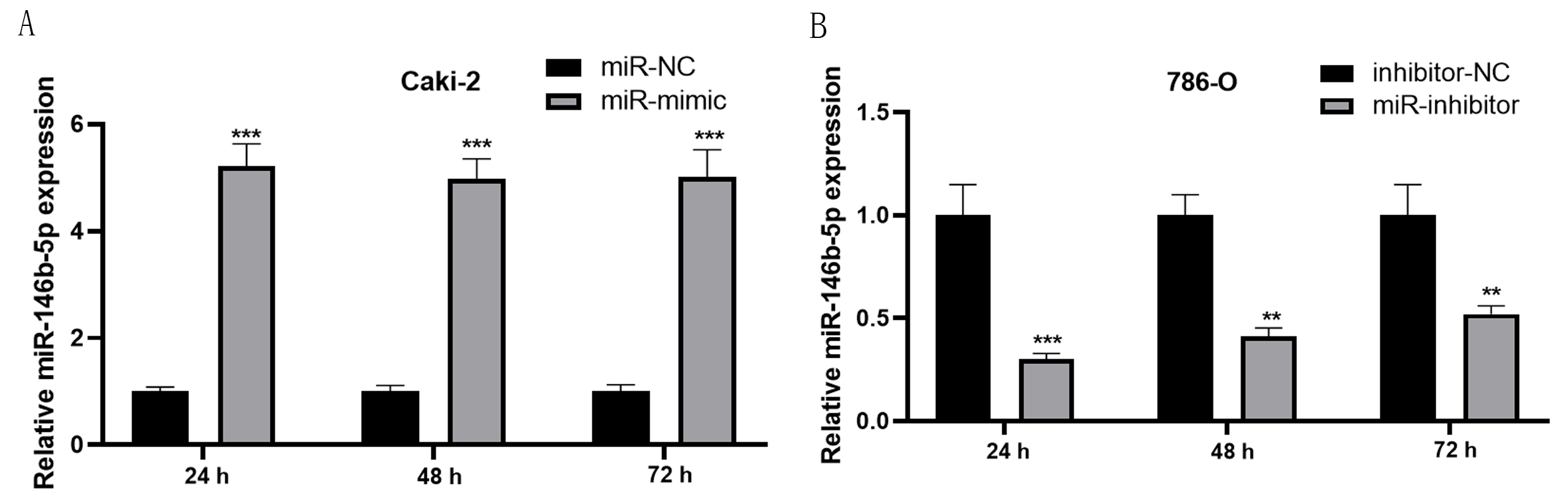

Supplement: Supplementary file 1 — Additional file 1. Figure S1 Correlation between transfection efficiency and transfection time. A-B: Transfection efficiency of miR-146b-5p mimic in Caki-2 cells or of miR-146b-5p inhibitor in 786-O cells at 24 h, 48 h and 72 h; All the above experiments were performed with 3 biological replicates; ** P<0.01 indicates significant difference, ***P<0.001 indicates extremely significant difference. [file 13008_2023_83_MOESM1_ESM.tif]
